# Food Waste among Young Adults: Behaviors, Barriers, and Opportunities for Intervention

**DOI:** 10.1016/j.cdnut.2025.107541

**Published:** 2025-09-03

**Authors:** Sara E Vandersip, Lindsey Smith Taillie, Anna H Grummon, Carmen E Prestemon, Alexandria E Reimold

**Affiliations:** 1 Department of Nutrition, Gillings School of Global Public Health, University of North Carolina, Chapel Hill, NC, United States; 2 Carolina Population Center, University of North Carolina, Chapel Hill, NC, United States; 3 Department of Pediatrics, Stanford University School of Medicine, Stanford, CA, United States; 4 Department of Health Policy, Stanford University School of Medicine, Stanford, CA, United States; 5 Department of Health Behavior, Gillings School of Global Public Health, University of North Carolina, Chapel Hill, NC, United States; 6 Department of Human Ecology, University of California, Davis, CA, United States

**Keywords:** environment, diet, food, and nutrition, perception, food loss and waste, college students

## Abstract

**Background:**

Reducing food waste is critical for protecting planetary and human health. Given that young adulthood is often a formative period for learning food management skills, young adults are a key demographic to study to better understand and intervene on food waste-related behaviors.

**Objectives:**

To describe food waste behaviors and barriers to reducing food waste among young adults.

**Methods:**

A cross-sectional online survey with a convenience sample (*n* = 2132) of United States young adults was conducted between November 2023 and December 2023. The survey measured self-reported food waste, difficulty reducing food waste, food waste-related behaviors, perceptions, intentions, and sociodemographic characteristics using items adapted from previous studies. Logistic regression was used to examine correlates of food waste.

**Results:**

Roughly 1-quarter (26%) of participants reported high food waste (≥30% of food wasted). Three-quarters of participants (77%) reported concern with food waste, and half (45%) reported being likely to reduce their food waste in the next 30 days. Non-Hispanic Black participants reported higher food waste than non-Hispanic White participants, whereas participants meeting financial needs with a little left over reported less food waste than those reporting comfortable financial situations (all *P* < 0.05). Barriers to reducing food waste included the inability to save food for later (25%), limited motivation to avoid food waste (18%), and not knowing how to avoid food waste (17%).

**Conclusions:**

Food waste varied by race and perceived financial situation. Barriers to reducing food waste could be addressed with environmental interventions that make it easier to save food for later and educational campaigns on why and how to reduce food waste.

## Introduction

Food waste is a critical problem to address for planetary and human health. In the United States, nearly 31% of total food production, valued at $382 billion, is wasted annually [1]. Food waste accounts for over 25% of the United States’ total freshwater consumption and 8% of global greenhouse gas emissions [2,3]. Thus, reducing food waste holds significant promise to mitigate climate change by conserving land and water resources and reducing global greenhouse gas emissions.

Reducing food waste could also provide important public health benefits. The United States’ annual food waste is the caloric equivalent of 149 billion meals or 1250 calories per person per day [1,4]. Repurposing palatable, safe food could increase food availability and food security [5]. Because the largest proportion (roughly 43%) of food wasted is produce, reducing food waste also has the potential to improve nutrition security, defined as consistent and equitable access to health-promoting foods [1,6].

The USDA and the United States Environmental Protection Agency have an ambitious food waste reduction goal of halving food waste by 2030 [7]. The residential sector, which is responsible for 40.8% of all food waste, presents an opportunity for reduction [1]. Within this sector, young adults are an important population to target to facilitate lasting food waste reduction, given that younger people tend to waste more food than older people [8]. Moreover, young adulthood is a critical developmental period in which individuals learn key food management skills—including food purchasing, preparation, storage, and disposal—all of which are behaviors that affect food waste [8]. These behaviors learned in young adulthood are often long-lasting, tracking throughout the lifespan [9].

Young adults are also at the forefront of social change [10]. Research shows that Generation Z (born between 1997 and 2012) and millennial (born between 1981 and 1996) generations prioritize climate change and environmental activism more than older generations, regardless of political party [11]. With food waste at the core of environmental issues, young adults may be especially motivated to adopt behaviors that reduce their personal food waste.

However, there are crucial gaps in our understanding of food waste among young adults. Although studies have examined behaviors associated with food waste among college students, few have studied these behaviors among young adults more generally (i.e., including those who are not current students), and none have documented food waste behaviors or targets for intervention with a large sample or on a national scale [[12], [13], [14], [15]]. Furthermore, there is limited evidence on how food waste among young adults varies by individual characteristics. Instead, existing studies have smaller sample sizes (*n* < 300), narrow geographic regions (i.e., 1 city or 1 university), and often report young adults as a homogenous group and are therefore less generalizable to diverse populations of young adults across the United States [12,[15], [16], [17]]. Therefore, the objective of this study was to explore self-reported food waste and barriers to reducing food waste among a large sample of young adults across the United States. We also aimed to examine young adults’ perceptions, intentions, and behaviors related to food waste. Finally, we examined sociodemographic correlates of self-reported food waste and perceived difficulty reducing food waste.

## Methods

### Participants

**FIGURE 1:**
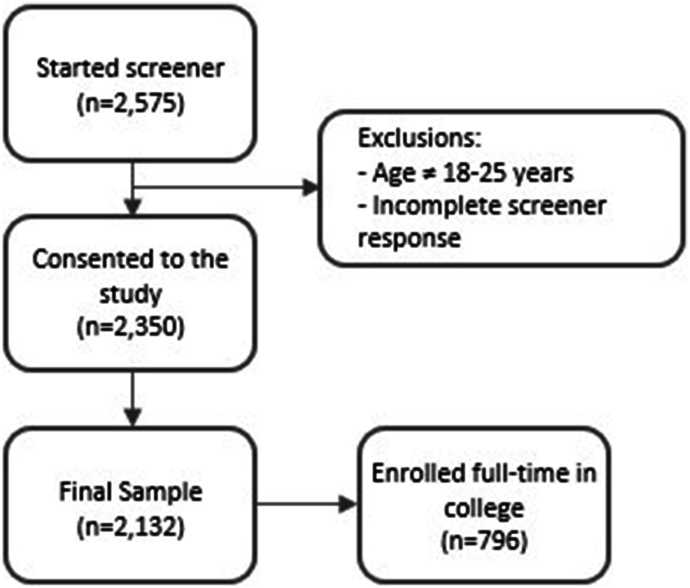
Participant flow chart.

Participants were recruited between November 2023 and December 2023 using the online survey research platform, CloudResearch Prime Panels. Of the 2575 participants who started the survey, 2350 consented to the study, and 2132 completed the study (Figure 1). The survey research platform used quota sampling to ensure that ≥25% of participants were currently enrolled full-time at colleges or universities. Participants were eligible if they were between 18 y and 25 y old.

### Procedures

Participants completed an online survey programmed using Qualtrics survey software. After providing online informed consent, participants completed an unrelated experiment as part of a parent study, then completed the food waste survey questions related to this study. After completing the survey, participants received compensation from the survey research platform in the form of reward points, gift cards, or cash. The study was determined to be exempt from further review by the University of North Carolina Institutional Review Board (#23-1785).

### Measures

The 2 primary outcomes of this study were dichotomous measures of self-reported personal food waste and perceived difficulty reducing food waste. We used the term outcome to describe the dependent variable, as is common for observational studies. The survey measured self-reported personal food waste using an item adapted from a previous study designed to measure food waste among adults of all ages in a nationally representative sample [18]. This item asked, “About what percent of all the food you acquire (i.e., purchase, obtain, or receive) is later thrown away? Give your best estimate.” with response options 0%, 10%, 20%, 30%, 40%, 50%, and >50%. Based on the frequencies of each response, we dichotomized food waste and defined high food waste as wasting ≥30% of personal food, representing the top quartile of responses. The survey assessed perceived difficulty reducing food waste with 1 item developed for this study that asked, “How difficult is it for you to avoid throwing away food?” with response options on a 5-point Likert scale (1 = not at all, 5 = a great deal). Based on the frequencies of each response, we dichotomized perceived difficulty and defined high perceived difficulty as reporting “3 = somewhat,” “4 = quite a bit,” or “5 = a great deal” of perceived difficulty in reducing food waste.

Next, the survey measured barriers to reducing food waste using 1 item [19]: “What makes it difficult to avoid throwing away food?” with the following response options: “I don’t know how to avoid throwing away food,” “I’m not motivated to avoid throwing away food,” “I don’t have a way to save food for later,” “I don’t have time to learn about repurposing food,” “I don’t have enough resources to repurpose food in my community,” “I don’t face any challenges in avoiding throwing away food,” “Other (with space to specify),” “Don’t know/unsure.” These response options capture barriers on the intrapersonal, community, and institutional levels of the socioecological framework and were selected based on frequency in the literature and expert opinion [20]. Participants could select all responses that applied [19].

The survey also measured concern with food waste, intentions to reduce food waste, and food waste prevention and reduction behaviors. The survey measured concern with food waste by asking, “Food waste refers to food that is fit for consumption but thrown away at the retail or consumption phases. To what extent, if at all, are you concerned about food waste in the United States?” using a 5-point Likert scale (1 = not at all concerned, 5 = very concerned) [12,21]. The survey measured intentions to reduce food waste with the question, “In the next 30 days, how likely are you to reduce the amount of food you throw out?” using a 5-point Likert scale (1 = very unlikely, 5 = very likely) [22]. The survey measured food waste prevention and reduction behaviors with 3 separate questions. The first asked, “How often do you shop for groceries?” with response options on a 6-point scale (1 = once a month or less, 6 = 3 or more times a week). The second asked, “Which of the following describes how you usually shop for groceries? Select all that apply,” with food-saving and food-wasting behavior response options [18] presented in a randomly assigned order. These included “I make a shopping list,” “ I check to see what is in my refrigerator and cupboards before I go shopping,” “I plan my meals before shopping,” “I estimate how much of various items I will need before shopping,” “I stick to my shopping list,” “I get tempted and buy appealing products” “I buy food in larger packages than I would prefer because of the way food is packaged,” “I buy more food than I need due to sale prices,” “I shop on an empty stomach,” and “None of these.” Participants could select all that applied. The third, multi-component question asked, “When you have excess or uneaten food, how often do you: *1*) dispose of it as trash, *2*) compost it, *3*) use containers to save it for later, and *4*) give it away to family or friends. Response options for each of the 4 behaviors listed were on a 5-point Likert scale (1 = very little or none, 5 = most or all). The extent to which participants engaged in each behavior was determined by combining the responses “4 = the majority” and “5 = most or all.”

The survey measured sociodemographic information using validated measures and measures adapted from previous studies, including gender [23], education and parental education [24], race and ethnicity [25], financial situation [26], political affiliation [27], and housing (using an item created for the survey). These sociodemographic characteristics were selected for inclusion based on a review of the food waste literature and expert opinion. Sociodemographic characteristics such as age, gender, race and ethnicity, education, and income are often associated with health behaviors, particularly food-related behaviors [28,29]. Thus, we wanted to explore how these factors might impact food waste behaviors. The full survey is in the supporting information (Supplemental File 1).

### Analysis

We used descriptive statistics to describe self-reported food waste, perceived difficulty reducing food waste, barriers to reducing food waste, concern with food waste, intentions to reduce food waste, existing food waste prevention and reduction behaviors, and sociodemographic characteristics. Next, we conducted a logistic regression to examine the relationship between sociodemographic characteristics (e.g., gender, education, race and ethnicity, financial situation, highest parental education, and political affiliation) and the binary outcomes *1*) high food waste and *2*) high perceived difficulty reducing food waste. We report results as average differential effects (i.e., differences in the predicted probabilities of having high food waste or high perceived difficulty by sociodemographic characteristics). All variance inflation factors were below 1.54, indicating no harmful multicollinearity. We used Stata/MP version 16.1, and analyses used 2-tailed statistical tests with a critical α of 0.05. The parent study this sample came from was preregistered on clinicaltrials.gov (NCT06119165) and AsPredicted.org (https://aspredicted.org/QXH_MR3); however, the analyses for this article were exploratory and not preregistered.

## Results

### Demographic characteristics

The mean age of participants in the study was 21.4 y (SD = 2.3 y). Half (50%) of respondents had a high school diploma, General Education Development test, or lower educational attainment (Table 1). Over two-thirds (68%) of respondents were White, 14% were Black, and 10% identified as Hispanic or Latino/a. Regionally, over one-third of participants were from the South (40%), followed by the Midwest (22%), the Northeast (20%), and the West (17%). In relation to financial health, 31% of participants reported living comfortably, 30% reported meeting their needs and having a little left, 31% reported just meeting basic expenses, and 8% reported not being able to meet their basic expenses. Just under half (43%) of participants reported parental education of high school, General Education Development, or lower educational attainment. Most respondents lived in a house (51%) or apartment (31%). Half (50%) of respondents were politically moderate, with slightly more liberal respondents (27%) than conservative (24%). Of all participants, 37% were enrolled full-time at a college or university.

**TABLE 1:** Participant demographic characteristics (*n* = 2132).^1^

	*n*	%
Average age (SD)	21.4	2.3
Gender		
Woman	1105	52
Man	973	46
Neither or prefer to self-describe	54	3
Education		
High school diploma, GED, or less	1057	50
Some college	614	29
Associate’s degree or more	461	22
Race and ethnicity		
Non-Hispanic White	1443	68
Hispanic, Latino, or Spanish	210	10
Non-Hispanic Asian	67	3
Non-Hispanic Black or African American	298	14
Non-Hispanic multi-racial	79	4
Non-Hispanic other racial identity	35	2
Region		
Midwest	478	22
Northeast	432	20
South	856	40
West	359	17
Financial situation		
Don’t meet basic expenses	157	8
Just meet basic expenses	638	31
Meet needs with a little left	619	30
Live comfortably	628	31
Parent 1 education		
No parents/guardian	75	4
High school diploma, GED, or less	909	43
Associate’s degree or some college	575	27
Bachelor's degree or more	572	27
Parent 2 education		
N/A - 1 parent/guardian	148	7
High school diploma, GED, or less	894	43
Associate’s degree or some college	504	25
Bachelor’s degree or more	511	25
Employment status		
Unemployed	782	37
Part-time or temporary work	588	28
Full-time work or >1 job	762	36
Housing		
House	1081	51
Apartment	660	31
University or sorority/fraternity housing	163	8
Other	228	11
Political ideology		
Moderate	1059	50
Conservative	503	24
Liberal	570	27
Enrolled full-time in college or university	796	37

Notes: other housing = townhouse, condominium, trailer, or other non-university housing.

Abbreviations: GED, General Education Development; SD, standard deviation; N/A, not applicable.

1 Missing demographic data ranged from 0% to 4%. – Not assessed.

### Self-reported food waste, perceived difficulty reducing food waste, and other outcomes

**FIGURE 2:**
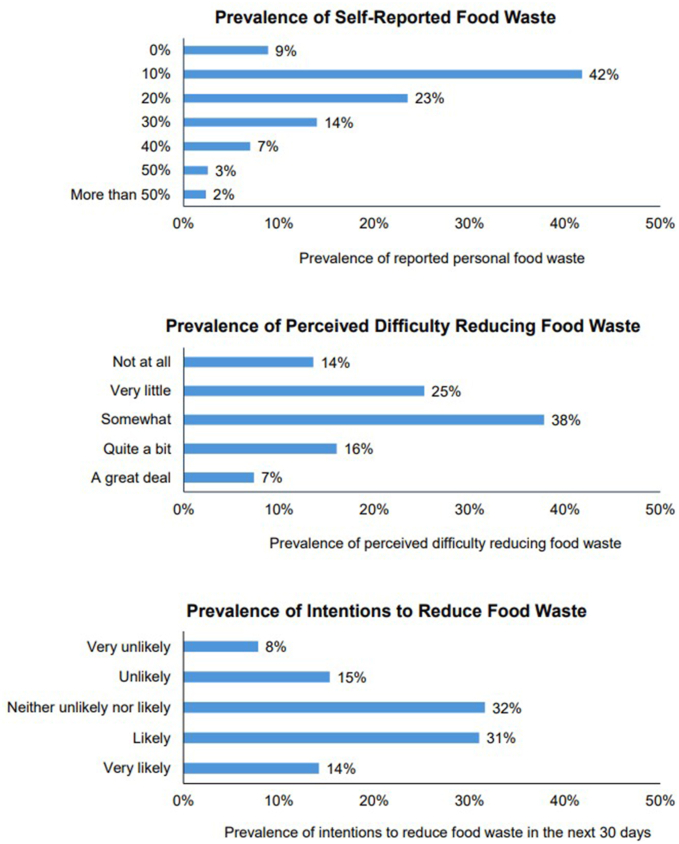
Prevalence of self-reported food waste, perceived difficulty reducing food waste, and intentions to reduce food waste.

One in 10 (9%) participants reported wasting no food, just under half (42%) reported wasting 10%, 1-quarter (23%) reported wasting 20%, 14% reported wasting 30%, 7% reported wasting 40%, 3% reported wasting 50%, and 2% of participants reported wasting >50% of their food (Figure 2). After dichotomizing this variable, roughly 1-quarter (26%) of participants reported high food waste (≥30% of food wasted). More than half (61%) of participants reported that barriers made reducing their food waste somewhat, quite a bit, or a great deal difficult. Most (77%) participants were concerned with food waste, but only 45% reported being likely or very likely to reduce their food waste in the next 30 d.

### Correlates of self-reported food waste

We found significant differences in self-reported food waste by race. We found that 39% of non-Hispanic Black participants reported high food waste and were 14% points more likely to report high food waste than non-Hispanic White participants, of which 23% reported high food waste [95% confidence interval (CI): 8%, 20%] (Table 2). We also found that 22% of participants who reported meeting their financial needs with a little left over reported high food waste and were 7% points less likely to report high food waste compared to those who reported having a comfortable financial situation, of which 28% reported high food waste (95% CI: ‒12%, ‒2%). Additionally, we observed that 22% of participants who reported not meeting basic expenses reported high food waste; however, due to a small cell size (*n* = 157), this result was not significantly different from other reported financial situations. We did not observe that gender, education, parental education, or political affiliation were associated with high food waste (all *P*’s > 0.05) (Table 2). Analyses also showed that there were no significant associations between sociodemographic characteristics and perceived difficulty reducing food waste (all *P*’s > 0.05) (Table 3).

**TABLE 2:** Correlates of self-reported food waste (*n* = 2042).^1^

	Reported high personal food waste		
	Average differential effect^2^	95% CI	*P* value
Gender			
Woman	Reference		
Man, %	0	‒4, 4	0.962
Neither or prefer to self-describe, %	3	‒11, 16	0.712
Education			
High school diploma, GED, or less	Reference		
Some college, %	‒4	‒9, 1	0.097
Associate’s degree or more, %	5	0, 11	0.063
Race and ethnicity			
Non-Hispanic White	Reference		
Hispanic or Latino, %	2	‒4, 9	0.491
Non-Hispanic Asian, %	7	‒5, 18	0.259
Non-Hispanic Black or African American, %	14	8, 20	<0.001
Non-Hispanic multi-racial, %	‒3	‒12, 7	0.577
Non-Hispanic other racial, % identity	‒2	‒16, 12	0.789
Financial situation			
Live comfortably	Reference		
Meet needs with a little left, %	‒7	‒12, ‒2	0.005
Just meet basic expenses, %	‒1	‒6, 4	0.800
Don’t meet basic expenses, %	‒7	‒14, 1	0.081
Highest parental education			
Bachelor’s degree or more	Reference		
Associate’s degree or some college, %	3	‒2, 8	0.193
High school diploma, GED, or less, %	5	0, 10	0.050
Political affiliation			
Moderate	Reference		
Conservative, %	2	‒3, 7	0.500
Liberal, %	‒1	‒6, 3	0.583

Note: Results presented in this table are from a multivariable logistic regression model with self-reported food waste as the dependent variable and demographic characteristics as the independent variables.

Abbreviations: CI, confidence interval; GED, General Education Development.

1 Analyses exclude 90 participants due to missing data.

2 Average differential effect = percentage point differences in the predicted probability of having high perceived difficulty.

**TABLE 3:** Correlates of perceived difficulty reducing food waste (*n* = 2042).^1^

	Reported high difficulty reducing food waste		
	Average differential effect^2^	95% CI	*P* value
Gender			
Woman	Reference		
Man, %	‒2	‒7, 2	0.263
Neither or prefer to self-describe, %	‒13	‒27, 2	0.089
Education			
High school diploma, GED, or less	Reference		
Some college, %	1	‒5, 6	0.787
Associate’s degree or more, %	5	‒1, 11	0.087
Race and ethnicity			
Non-Hispanic White	Reference		
Hispanic or Latino, %	3	‒4, 10	0.381
Non-Hispanic Asian, %	8	‒3, 20	0.157
Non-Hispanic Black or African American, %	‒4	‒11, 2	0.190
Non-Hispanic multi-racial, %	‒4	‒15, 8	0.530
Non-Hispanic other racial identity, %	2	‒15, 18	0.844
Financial situation			
Live comfortably	Reference		
Meet needs with a little left, %	1	‒5, 6	0.794
Just meet basic expenses, %	3	‒2, 9	0.244
Don’t meet basic expenses, %	7	‒1, 16	0.085
Highest parental education			
Bachelor’s degree or more	Reference		
Associate’s degree or some college, %	2	‒4, 8	0.479
High school diploma, GED, or less, %	3	‒3, 9	0.277
Political affiliation			
Moderate	Reference		
Conservative, %	2	‒4, 7	0.509
Liberal, %	0	‒5, 5	0.925

Note: Results presented in this table are from a multivariable logistic regression model with perceived difficulty reducing food waste as the dependent variable and demographic characteristics as the independent variables.

Abbreviations: CI, confidence interval; GED, General Education Development.

1 Analyses exclude 90 participants due to missing data.

2 Average differential effect = percentage point differences in the predicted probability of having high perceived difficulty.

### Top reported barriers, grocery shopping behaviors, and food-saving techniques

**FIGURE 3:**
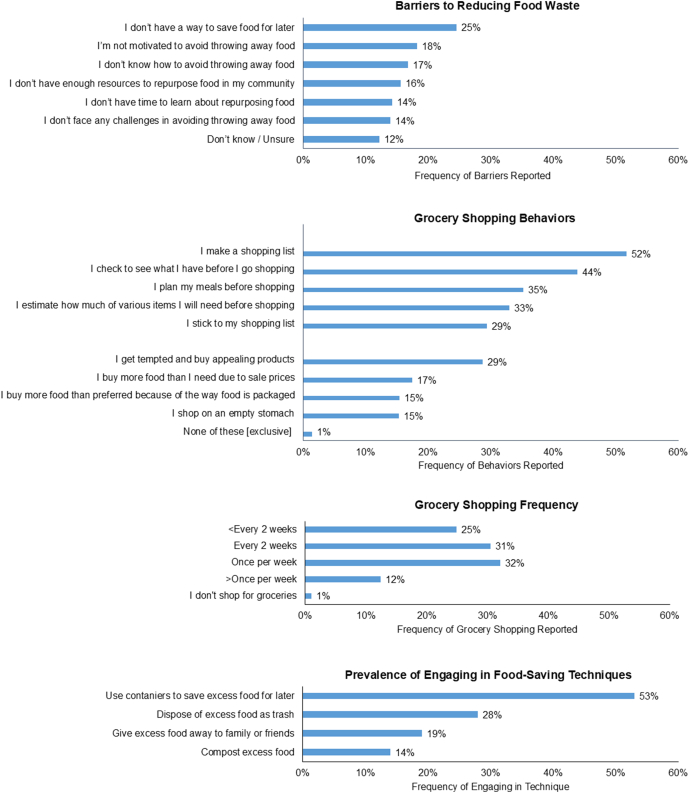
Prevalence of barriers, grocery shopping behaviors, and food-saving techniques.

The top 4 reported barriers to reducing food waste were not having a way to save food for later (25%), not being motivated to avoid throwing away food (18%), not knowing how to avoid throwing away food (17%), and not having enough community resources to repurpose food (16%) (Figure 3). Grocery shopping behaviors known to be associated with preventing food waste, or food-saving behaviors, were more prevalent than grocery shopping behaviors known to be associated with increasing food waste, or food-wasting behaviors. The most common food-saving behaviors were making a grocery list (52%) and checking food available at home before grocery shopping (44%). The most prevalent food-wasting grocery shopping behaviors were feeling tempted to buy appealing impulse products (29%) and buying more food than needed due to sale prices (17%). The most common grocery shopping frequencies were once a week (32%) and once every 2 wk (31%). Engagement in food-saving varied depending on the technique. Roughly half (53%) of participants reported using containers to save excess food for later, 19% said they give away excess food, and 14% said they compost excess food.

## Discussion

In this exploratory study with a national sample of United States young adults, 1-quarter (26%) of participants reported high food waste (≥30% of food wasted), and 3-quarters of participants (77%) reported concern with food waste; however, less than half of the participants (45%) reported being likely to reduce their food waste in the next 30 d. Food waste varied by sociodemographic characteristics such that non-Hispanic Black participants reported higher food waste than non-Hispanic White participants, whereas participants who met their financial needs and had a little left over reported less food waste than those who reported comfortable financial situations (all *P* < 0.05). Participants reported that the inability to save food for later (25%), limited motivation to avoid food waste (18%), and not knowing how to avoid food waste (17%) were barriers to reducing their personal food waste.

The average objective measure of United States household food waste, most often measured by the weight of food purchased and wasted, is roughly 32% of household food [30]. The discrepancy in the self-reported food waste in this study and more objective measures aligns with existing literature showing that self-reported food waste is consistently lower than objective measures [12,18,[31], [32], [33]]. However, the self-reported food waste in our sample of young adults was slightly higher than the average self-reported food waste in other United States studies with samples of adults of all ages. For example, a study with a nationally representative sample of United States adults (18 y and older) found that 90% of participants reported wasting ≤20% of their household’s food [18], whereas in our study, only 75% of participants reported wasting ≤20% of their personal food. These findings are consistent with the inverse relationship between age and food waste found in existing literature on self-reported food waste [8,12].

Similar to other studies [12,18], the majority of young adults in this study (77%) reported being concerned with food waste in the United States; however, most (55%) did not have intentions to reduce their own food waste. This suggests that concern with food waste does not necessarily translate to intentions to reduce food waste among young adults, perhaps because of the unique barriers that young adults face when it comes to reducing food waste. In this study, almost two-thirds (61%) of participants reported that reducing their food waste was difficult. The most frequently reported barriers to reducing food waste were not having a way to save food for later, not being motivated to avoid throwing away food, not knowing how to avoid throwing away food, and not having community resources to repurpose food. These barriers could interfere with young adults’ intentions to reduce food waste, regardless of their level of concern. The discrepancy between concern with food waste and intentions to reduce food waste may also be related to imprecise perceptions of personal food waste. Because most participants self-reported wasting only 10‒20% of their food, substantially less than objective measures [12,18,30], participants may have had lower intentions to reduce food waste based on a belief that their food waste was already low. Another possible explanation for participants’ low intentions to reduce food waste could be that they have limited awareness of the consequences of wasting food, as existing literature shows that awareness of consequences and environmental knowledge are associated with increased intentions to reduce food waste [34].

Young adults reported infrequently engaging in several food-saving behaviors, including planning meals before shopping (35%), estimating amounts of grocery items needed before shopping (33%), and sticking to a shopping list (29%). Similarly, ∼15‒30% of participants reported engaging in food-wasting shopping behaviors such as being tempted to buy unplanned products and buying more food than needed due to sale prices. Infrequent food-saving behaviors may be due to limited food preparation and storage skills [35], and though limited, the food-wasting shopping behaviors may be due to higher levels of impulsivity among young adults [35]. These behaviors are likely also due to barriers experienced by young adults.

The most frequently reported barrier to reducing food waste was not having a way to save food for later, and a substantial number of participants also reported not having enough community resources. This mirrors existing research, finding a lack of infrastructure for food donation and recirculation in communities as a key barrier to reducing food waste [12,[36], [37], [38]]. These results highlight the specific structural barriers that future food waste interventions could target. For example, expanding community resources for storing and donating food could improve food recirculation among young adults [39]. Additionally, 1 of the most supported food waste solutions among United States adults is to make it easier to donate food [40], indicating that redistributing excess or leftover food may be an effective strategy for reducing food waste on college campuses [39]. Therefore, resources that foster food recovery networks and offer storage space, such as food pantries, community fridges, or community gardens, could provide a solution to the barriers young adults face [41]. Improving food donation and recirculation among young adults could also bolster a sense of community, which is in turn associated with increased intentions to reduce food waste [34]. These findings emphasize the importance of specifically tailored, place-based policies for reducing food waste [42,43].

Many young adults in our study also reported not being motivated and not knowing how to reduce food waste. These results again highlight a potential component of food waste that future interventions could target. Previous research indicates that tailored educational campaigns can increase awareness and self-efficacy with food-saving habits among young adults [44].

We found that 39% of non-Hispanic Black participants reported high food waste and were 14% points more likely to report high food waste than non-Hispanic White participants, of which 23% reported high food waste. These findings may reflect additional barriers that young, Black adults often experience, especially in the context of structural racism and racial inequities in the United States. The unequal allocation of goods, services, and investment in Black communities results in socioeconomic and environmental inequities, which could also drive food waste [45]. Future studies should examine the relationships between sociodemographic characteristics, food waste, and barriers to reducing food waste.

Additionally, we found that 22% of participants who reported meeting their financial needs with a little left over reported high food waste and were 7% points less likely to report high food waste compared to those reporting comfortable financial situations, of which 28% reported high food waste. The financial cost of food waste is a top concern for many young adults, especially those raised in low-income households [12]. It is possible that participants in more secure financial situations were less worried about the cost of food waste, and were therefore more likely to waste food [12,18]. However, those who reported they “just meet basic expenses” or “don’t meet basic expenses” were not more likely to report high food waste compared to those living comfortably, suggesting more complicated associations between perceived financial situation and food waste among young adults that should be further assessed in future research.

None of the sociodemographic characteristics we assessed were associated with perceived difficulty reducing food waste in our sample of young adults. These results are consistent with a previous study positing that sociodemographic characteristics do not play a major role in explaining the causes of consumer food waste above and beyond psychographic factors like motivation, awareness, knowledge, and capabilities [46]. Exploring how and why barriers and behaviors related to food waste are associated with perceived difficulty reducing food waste should be further evaluated in future research.

### Strengths and limitations

This study adds to the small body of literature on food waste among young adults. Strengths of this study include the large and diverse sample that surveyed participants with various gender identities, racial-ethnic identities, geographical regions, financial situations, educational attainment, and political affiliation. Another strength is the assessment of a wide range of behaviors, perceptions, and intentions related to food waste. Furthermore, the primary outcome of self-reported food waste was developed from a previously published measure and reviewed by experts. However, the use of an online panel may limit generalizability, as it is possible that participants in online panels may differ from nonpanelists with regard to sociodemographic characteristics and food shopping, saving, and wasting behaviors. Limitations of this study also include how the survey described “food waste” and “throwing away food.” Participants may have differed in their subjective understandings of these terms, especially because the survey did not define the state of food being wasted (i.e., perished, past expiration date, leftovers, kitchen scraps, and more). Furthermore, we asked participants about personal food waste, but we did not survey whether participants were the primary food shoppers or preparers for themselves. Additionally, the survey measure of the extent to which participants engage in food-saving behaviors was worded to determine frequency, whereas the response options assessed quantity, which may have influenced responses. There could also be potential confounding variables that influence the results of this analysis, such as the barriers and behaviors related to food waste discussed earlier. Lastly, 218 participants consented to the survey but did not complete it. Though this is a small proportion of the sample, it is possible that this biased the results.

In conclusion, the results of this exploratory study suggest that barriers to reducing food waste are prevalent among young adults, and perceived difficulty reducing food waste is not significantly different across various demographic groups. At the intrapersonal level, educational interventions such as tailored campaigns, websites, applications, and courses at universities or workplaces may increase knowledge and motivation among young adults to avoid throwing away food. At the community level, establishing more food pantries, community kitchens, and promoting the use of food donation applications may increase young adults’ ability and motivation to repurpose food. Finally, at the institutional level, policies that require food-serving institutions (e.g., restaurants, workplace and university dining halls, hospitals) to offer takeout containers and incorporate more refrigeration storage spaces may address common barriers to food waste reduction.

## Author contributions

The authors’ responsibilities were as follows – SEV, LST, AER: conceptualization; SEV, CEP: data curation; SEV, AHG, CEP, LST, AER: formal analysis, investigation, methodology, and writing – review and editing; CEP: project administration; LST, AHG: supervision; SEV: visualization; SEV: writing – original draft; and all authors: read and approved the final manuscript.

## Data availability

All datasets and metadata described in the manuscript, codebook, and analytic code are available from the University of North Carolina Dataverse database (https://dataverse.unc.edu/dataverse/foodwaste).

## Funding

This work was supported by the Wellcome Trust (grant ID # 216042/Z/19/Z), received by LST. The funders had no role in study design, data collection and analysis, decision to publish, or preparation of the manuscript.

## Conflict of interest

LST, AHG, and CEP received salary support from the Wellcome Trust (website: https://wellcome.org/). All other authors report no conflicts of interest.
