# Supplementary material for: Food Waste among Young Adults: Behaviors, Barriers, and Opportunities for Intervention
Source: Curr Dev Nutr. 2025 Sep 3;9(10):107541. doi: 10.1016/j.cdnut.2025.107541 (PMC12538431; doi:10.1016/j.cdnut.2025.107541)
Supplement: Multimedia component 1 [file mmc1.docx]

**“Food Waste Among Young Adults: Behaviors, Barriers, and Opportunities for Intervention”**

**Supplementary File 1. Full Survey Codebook**

**Sara Vandersip**

| **Construct**  **Question ID**  **(source)** | **Measure** | **Response options (where applicable)** |
| --- | --- | --- |
| **CONSENT** | | |
| **consent** | **[INSERT CONSENT]**  **[Page Break]** |  |
| **SCREENER** | | |
| **scr_intro** | Welcome! This survey will ask you questions about your experience with purchasing and eating food.  [Page Break] |  |
| **age** | How old are you? Enter your age in years. | Open ended  [Add skip logic – if under 18 y/o or older than 25 y/o skip to end, restrict 0-99] |
| **gender (**[**developed using guidelines from the National Academies report**](https://nap.nationalacademies.org/read/26424/chapter/10#121)**)** | Select the option that best describes your gender. | 1 = Woman  2 = Man  3 = Neither woman nor man  4 = Prefer to self-describe: _______  5 = Prefer not to say |
| **race**  **(**[**U.S. Census Bureau, combined race/ethnicity question**](https://www.census.gov/newsroom/blogs/random-samplings/2024/04/updates-race-ethnicity-standards.html)**)** | What is your race and/or ethnicity? Select all that apply. | 1 = White  2 = Hispanic, Latino, or Spanish  3 = Black or African American  4 = Asian  5 = American Indian or Alaska Native  6 = Middle Eastern or Northern African  7 = Native Hawaiian or Other Pacific Islander  8 = Some other race or ethnicity (please specify): _____ |
| **education (**[**2022 American Community Survey, modified**](https://www.census.gov/acs/www/about/why-we-ask-each-question/education/)**)** | What is the highest level of school you have completed? | 1 =Less than high school or U.S. high school equivalent (GED)  2 =High school diploma or U.S. high school equivalent (GED)  3 =Some college  4 =Associate’s degree  5 =4-year college degree  6 =Graduate degree |
| **college** | Are you currently enrolled **full-time** at a college or university? | 1 = Yes  0 = No  [quota set to at least 25% “yes”] |
| **FOOD PLANNING AND BEHAVIOR** | | |
| **Behav_Intro** | The next few questions ask about how you plan, shop for, and cook food.  [page break] |  |
| **groceries1** | Where do you typically get groceries from? Select up to three.  [page break] | (Can select up to 3)  1 = [Grocery stores](https://www.foodindustry.com/articles/top-10-grocers-in-the-united-states-2019/) (e.g., Kroger, Albertsons, Publix, H-E-B, ALDI)  2 = On-campus grocers/markets  3 = Convenience stores or gas stations  4 = Dollar stores (e.g., Dollar General, Family Dollar, Dollar Tree)  5 = Warehouse club stores (e.g., Costco, BJs, Sam’s Club)  6 = Mass merchandisers (e.g., Walmart, Target)  7 = Farmer’s markets  8 = Ethnic or specialty stores  9 = Food bank or food donations  10 = Other: __________  0 = I don’t shop for groceries **[exclusive]** |
| **groceries2** | How often do you shop for groceries?  **[do not display if groceries1=0]** | 1 = Once a month or less  2 = Once every three weeks  3 = Once every two weeks  4 = Once a week  5 = Twice a week  6 = Three or more times a week |
| **shopping (**[**Neff**](https://doi.org/10.1371/journal.pone.0127881.s006)**)** | Which of the following describe how you usually shop for groceries? Select all that apply.  **[do not display if groceries1=0)]**. | [randomize order]  1 = I make a shopping list  2 = I check to see what is in my refrigerator and cupboards before I go shopping  3 = I plan my meals before shopping  4 = I estimate how much of various items I will need before shopping  5 = I stick to my shopping list  6 = I get tempted and buy appealing products  7 = I buy food in larger packages than I would prefer because of the way food is packaged  8 = I buy more food than I need due to sale prices  9 = I shop on an empty stomach  10 = None of these **[exclusive]** |
| **SELF-ASSESSMENT OF FOOD WASTE** | | |
| **self_FW (Adapted from** [**Neff**](https://doi.org/10.1371/journal.pone.0127881.s006)**)** | About what percent of all the food you acquire (i.e., purchase, obtain, or receive) is later thrown away? Give your best estimate. | 0 = 0%  1 = 10%  2 = 20%  3 = 30%  4 = 40%  5 = 50%  6 = More than 50% |
| **PERCEPTIONS OF FOOD WASTE** | | |
| **perceptions_intro** | The next few questions will ask about excess or uneaten food. This refers to when you bought or prepared more food than you can use or eat. |  |
| **uneaten_food** | When you have excess or uneaten food, how often do you:  Dispose it as trash  Compost it  Use containers to save it for later  Give it away to family or friends | (list as a matrix)  1 = Very little or none  2 = A bit  3 = Some but not more than half  4 = The majority  5 = Most or all |
| **BARRIERS AND CHALLENGES TO REDUCING FOOD WASTE** | | |
| **difficult** | How difficult is it for you to avoid throwing away food? | (Likert scale)  1=Not at all  2=Very little  3=Somewhat  4=Quite a bit  5=A great deal |
| **barrier**  **(Adapted from** [**Filho**](https://www.scopus.com/record/display.uri?eid=2-s2.0-85098870416&doi=10.1080%2f13504509.2020.1865474&origin=inward&txGid=f2c8cab71f1263fa4fcdec7934621aed)**)** | What makes it difficult to avoid throwing away food? | 1 = I don’t know how to avoid throwing away food  2 = I’m not motivated to avoid throwing away food  3 = I don’t have a way to save food for later  4 = I don’t have time to learn about repurposing food  5 = I don’t have enough resources to repurpose food in my community  6 = Other: ______  7 =I don’t face any challenges in avoiding throwing away food [exclusive]  8 = Don’t know / Unsure [exclusive]  [program to allow multiple responses] |
| **intentions**    Intentions to reduce food waste  Adapted from Malek et al (2019) | In the next 30 days, how likely are you to reduce the amount of food you throw out?    [page break] | 1 = Very unlikely  2 = Somewhat unlikely  3 = Neither unlikely nor likely  4 = Somewhat likely  5 = Very likely |
| **concern (**[**Nikolaus,**](https://pubmed.ncbi.nlm.nih.gov/30063960/) [Harvard](https://www.hsph.harvard.edu/nutritionsource/sustainability/food-waste/)**)** | Food waste refers to food that is fit for consumption but thrown away at the retail or consumption phases. To what extent, if at all, are you concerned about food waste in the US? | 1 = Not at all concerned  2 = Slightly concerned  3 = Somewhat concerned  4 = Moderately concerned  5 = Very concerned |
| **DEMOGRAPHICS** | | |
| **dem_Intro** | We are asking the next questions to better understand who took our survey.  [Page Break] |  |
| **parental_educ1 (adapted from** [**2022 American Community Survey**](https://www.census.gov/acs/www/about/why-we-ask-each-question/education/)**)** | First, we would like to ask about your two primary parents/guardians.  What is the highest level of education your parent/guardian #1 has received? | 0 = I don’t have a parent/guardian(s) [add skip logic - skip to work]  1 = Elementary school (grades 1 through 4)  2 = Middle school (grades 5 through 8)  3 = Some high school (no diploma)  4 = High school diploma  5 = GED or alternative credential  6 = Some college (no degree)  7 = Associate’s degree/technical school/apprenticeship  8 = Bachelor’s degree  9 = Postgraduate (e.g., Master’s, PhD) / Professional degree (e.g., JD) |
| **parental_educ2 (adapted from** [**2022 American Community Survey**](https://www.census.gov/acs/www/about/why-we-ask-each-question/education/)**)** | What is the highest level of education your parent/guardian #2 has received? | 0 = N/A  1 = Elementary school (grades 1 through 4)  2 = Middle school (grades 5 through 8)  3 = Some high school (no diploma)  4 = High school diploma  5 = GED or alternative credential  6 = Some college (no degree)  7 = Associate’s degree/technical school/apprenticeship  8 = Bachelor’s degree  9 = Postgraduate (e.g., Master’s, PhD) / Professional degree (e.g., JD) |
| **finsit**      Williams VF, Smith AA, Villanti AC, et al. Validity of a Subjective Financial Situation Measure to Assess Socioeconomic Status in US Young Adults. *Journal of Public Health Management and Practice*. 2017;23(5):487-495. | Considering your own income and the income from any other people who help you, how would you describe your overall financial situation? | 4 = Live comfortably  3 = Meet needs with a little left  2 = Just meet basic expenses  1 = Don’t meet basic expenses |
| **work** | What is your current work situation? | 1 = Unemployed  2 = Unemployed but not seeking work (e.g., student, retired, disabled, unpaid primary caregiver)  3 = Part-time or temporary work  4 = Full-time work  5 = Two or more part-time or full-time jobs |
| **residence** | What type of residence do you live in? | 1 = House  2 = Apartment  3 = Townhouse  4 = Condominium  5 = Trailer  6 = Dormitory  7 = University apartment  8 = Sorority/fraternity house  9 = Other university housing: _______  10 = Other non-university housing: _______ |
| **politics (adapted from** [**Pew Research Center**](https://www.pewresearch.org/internet/wp-content/uploads/sites/9/2013/04/SurveyQuestions_CivicEngagement.pdf)**)** | In general, how would you describe your political views? | 1 = Very conservative  2 = Conservative  3 = Moderate  4 = Liberal  5 = Very liberal |
| Closure | Anything else you want to tell us? | [Free response] |

**References**

1. Morata MP, González-Santana RA, Blesa J, Frígola A, Esteve MJ. A study of the habits and food waste production of young university students. *Nutricion Hospitalaria*. 2020;37(2):349-358. doi:[10.20960/nh.02833](https://doi.org/10.20960/nh.02833)

2. Krupp B, Gersey J, Lebo F. Campus Plate: Connecting Students on College Campuses to Reduce Food Waste and Food Insecurity. In: ; 2022:172-177. doi:[10.1145/3538641.3561506](https://doi.org/10.1145/3538641.3561506)

3. Null DC, Asirvatham J. College students are pro-environment but lack sustainability knowledge: a study at a mid-size Midwestern US university. *International Journal of Sustainability in Higher Education*. 2023;24(3):660-677. doi:[10.1108/IJSHE-02-2022-0046](https://doi.org/10.1108/IJSHE-02-2022-0046)

4. Wiriyaphanich T, Guinard JX, Spang E, et al. Food Choice and Waste in University Dining Commons-A Menus of Change University Research Collaborative Study. *Foods*. 2021;10(3):577. doi:[10.3390/foods10030577](https://doi.org/10.3390/foods10030577)

5. Avenue 677 Huntington, Boston, Ma 02115. Food Waste. The Nutrition Source. Published April 26, 2017. Accessed June 19, 2023. <https://www.hsph.harvard.edu/nutritionsource/sustainability/food-waste/>

6. Musicus AA, Amsler Challamel GC, McKenzie R, Rimm EB, Blondin SA. Food Waste Management Practices and Barriers to Progress in U.S. University Foodservice. *Int J Environ Res Public Health*. 2022;19(11):6512. doi:[10.3390/ijerph19116512](https://doi.org/10.3390/ijerph19116512)

7. Börühan G, Ozbiltekin-Pala M. Food waste management: an example from university refectory. *British Food Journal*. 2021;124(1):293-313. doi:[10.1108/BFJ-09-2020-0802](https://doi.org/10.1108/BFJ-09-2020-0802)

8. Frank LB, Finkbinder EM, Powell VS. “Free Food on Campus!”: A Novel Use of Instructional Technology to Reduce University Food Waste and Feed Hungry Students. *Journal of Hunger and Environmental Nutrition*. Published online 2021. doi:[10.1080/19320248.2020.1850389](https://doi.org/10.1080/19320248.2020.1850389)

9. Gabriel A, Rombach M, Wieser H, Bitsch V. Got waste: Knowledge, behavior and self-assessment on food waste of university students in Germany. *International Food and Agribusiness Management Review*. 2021;24(6):951-970. doi:[10.22434/IFAMR2020.0145](https://doi.org/10.22434/IFAMR2020.0145)

10. Leal Filho W, Lange Salvia A, Davis B, Will M, Moggi S. Higher education and food waste: assessing current trends. *International Journal of Sustainable Development and World Ecology*. 2021;28(5):440-450. doi:[10.1080/13504509.2020.1865474](https://doi.org/10.1080/13504509.2020.1865474)

11. Richardson R, Prescott MP, Ellison B. Impact of plate shape and size on individual food waste in a university dining hall. *Resources, Conservation and Recycling*. 2021;168:105293. doi:[10.1016/j.resconrec.2020.105293](https://doi.org/10.1016/j.resconrec.2020.105293)

12. Davison N, Young W, Ross A, Cockerill T, Rajput S. Investigating the Impacts of Behavioural-Change Interventions and COVID-19 on the Food-Waste-Generation Behaviours of Catered Students in the UK and India. *Sustainability (Switzerland)*. 2022;14(9). doi:[10.3390/su14095486](https://doi.org/10.3390/su14095486)

13. US EPA O. Preventing Wasted Food At Home. Published April 18, 2013. Accessed June 19, 2023. <https://www.epa.gov/recycle/preventing-wasted-food-home>

14. Kaur P, Dhir A, Talwar S, Alrasheedy M. Systematic literature review of food waste in educational institutions: setting the research agenda. *International Journal of Contemporary Hospitality Management*. 2020;33(4):1160-1193. doi:[10.1108/IJCHM-07-2020-0672](https://doi.org/10.1108/IJCHM-07-2020-0672)

15. Picardy JA, Ghezzi S, Bilodeau R. Teaching Sustainability Practice through Service Learning: A Case Study of Reducing Food Waste. *Sustainability and climate change*. 2021;14(1):55-59. doi:[10.1089/scc.2020.0048](https://doi.org/10.1089/scc.2020.0048)

16. Understand College Campus and Student Body Size – BigFuture | College Board. Accessed June 19, 2023. <https://bigfuture.collegeboard.org/plan-for-college/college-basics/types-of-colleges/understand-college-campus-student-body-size>

17. Ozanne LK, Ballantine PW, McMaster A. Understanding Food Waste Produced by University Students: A Social Practice Approach. *Sustainability (Switzerland)*. 2022;14(17). doi:[10.3390/su141710653](https://doi.org/10.3390/su141710653)

18. Radandt NE, Corbridge T, Johnson DB, Kim AS, Scott JM, Coldwell SE. Validation of a Two-Item Food Security Screening Tool in a Dental Setting. *J Dent Child (Chic)*. 2018;85(3):114-119.

19. Nikolaus CJ, Nickols-Richardson SM, Ellison B. Wasted food: A qualitative study of U.S. young adults’ perceptions, beliefs and behaviors. *Appetite*. 2018;130:70-78. doi:[10.1016/j.appet.2018.07.026](https://doi.org/10.1016/j.appet.2018.07.026)

20. Neff RA, Spiker ML, Truant PL. Wasted Food: U.S. Consumers’ Reported Awareness, Attitudes, and Behaviors. Wiley AS, ed. *PLoS ONE*. 2015;10(6):e0127881. doi:[10.1371/journal.pone.0127881](https://doi.org/10.1371/journal.pone.0127881)

21. Team FE. Who are the top 10 Grocers in the United States? FoodIndustry.Com. Published June 7, 2022. Accessed June 27, 2023. <https://www.foodindustry.com/articles/top-10-grocers-in-the-united-states-2019/>
